# Supra-costal tubeless percutaneous nephrolithotomy is not associated with increased complication rate: a prospective study of safety and efficacy of supra-costal versus sub-costal access

**DOI:** 10.1186/s12894-018-0429-1

**Published:** 2018-12-11

**Authors:** Meng-Yi Yan, Jesun Lin, Heng-Chieh Chiang, Yao-Li Chen, Pao-Hwa Chen

**Affiliations:** 1 From the Division of Urology, Department of Surgery, Changhua Christian Hospital, 135, Nanxiao St., Changhua City, Changhua County 500 Taiwan; 2 From the Transplant Medicine and Surgery Research Center, Changhua Christian Hospital, Changhua, Taiwan; 3 School of Medicine, Kaohsiung Medical University, Kaohsiung, Taiwan

**Keywords:** Tubeless PCNL, Surgical complications, Supra-costal, Nephrolithotomy

## Abstract

**Background:**

To assess the morbidities of tubeless percutaneous nephrolithotomy (PCNL) using supra-costal access and re-evaluate traditional concept of increased complications with supra-costal access.

**Methods:**

From January 2010 to December 2014, a single surgeon performed 118 consecutive one-stage fluoroscopic guided PCNL’s for complex renal and upper ureteral stone. Our definition for complex renal stone is defined as partial or complete staghorn stone, multiple renal stones in more than 2 calyxes, obstructive uretero-pelvic stone > 2 cm, and a renal stone in single functional kidney. Inclusion criteria include: staghorn stones, renal calculi > 2 cm in diameter, upper ureteral stone > 1.5 cm in diameter. Exclusion criteria for tubeless PCNL include: significant bleeding or perforation of the collecting system, large residue stone, multiple PCNL tract and obstructive renal anatomy. Morbidity, operation time, analgesia requirement, length of hospital stay, stone- free rate, were analyzed.

**Results:**

Of the 118 consecutive PCNL, eighty-six patients underwent tubeless PCNL (56 supra-costal and 30 sub-costal) and included in our prospective follow-up period. The mean age, operation side, stone locations were similar. The male to female ratio is higher in supra-costal than sub-costal. Large renal stones and staghorn stones makes up for most patients (supra-costal: 75%, sub-costal: 80%). The stone–free rate of supra-costal group was 59% (33/56) and in sub-costal group was 50% (15/30). The operative times, length of stay, post-op analgesic use, hematocrit change was similar in both groups. The overall complication rate is 6% [supra-costal (1/56), sub-costal (4/30)] with the majority being infectious complications.

**Conclusions:**

Supra-costal access above 12th rib during tubeless PCNL is safe and effective procedure and is not associated with higher incidence of post-op complications in experience hands.

## Background

Since Fernstrom and Johansson performed the first percutaneous nephrolithotomy (PCNL) was performed in 1976, endourological approach has taken an increase role in management of complex urinary calculi [1, 2]. In the recent periods, minimally invasive surgical procedures using advanced instruments and techniques have gradually replaced open surgery for treating large, complex renal and upper ureteral stones. According to the American Urological Association (AUA) guidelines and European Association of Urology (EAU) guideline on urolithiasis, percutaneous nephrolithotripsy (PCNL) is the first-line treatment for renal staghorn stones and renal stones larger than 2 cm. During standard PCNL, the placement of a nephrostomy tube after the operation is a common practice which provides hemostasis, adequate drainage and retaining access for future endoscopic procedures. In selected cases with minimal bleeding and those not needing subsequent percutaneous access, tubeless PCNL has been found to be a safe and effective practice. In previous studies, tubeless PCNL has been showed to reduce hospital stay and post-operative pain compared to conventional nephrostomy tube placement [3–10].

According to results of Hopper & Yakes’ study, intercostal percutaneous approach between the 11th and 12th rib into the collecting system would result in lung injury in 14% on the left and 29% in right side. [11] If the puncture is in the 10th–11th rib intercostal space, lung injury is expected in 86% on the left and 93% right side. The key factor in a successful PCNL surgery is selecting the appropriate calyx to gain access to the collecting system. In certain situation such as large or complicated renal stone, an upper pole access will ensure better stone free rate. In most cases, supra-costal approach (intercostal space between 11th and 12th rib) will provide the easiest and the most direct access of the upper calyx in the collecting system. Therefore for large complicated stones, an supra-costal approach is necessary to obtain the best stone free rate [12]. On the other hand, the increased risk of injury to the surrounding organs (pleura, lung, spleen or liver) reported in previous literatures of supra-costal approach is strongly discouraged [13, 14].

In recent studies, tubeless PCNL offers the potential advantages of decreased post-operative pain leading to decrease analgesic use and hospital stay without increasing the complications [4, 5, 8–10, 15, 16]. Since there is a very limited literatures discussing tubeless PCNL using supra-costal approach, the questions of increased complication and morbidities associated with supra-costal approach when compared to sub-costal (below 12th rib) approach is still debatable [4, 15]. Therefore, we set out to prospectively analyze the morbidity associated with supra-costal and sub-costal approach using tubeless percutaneous nephrolithotomy.

## Methods

After obtaining Institutional review board (IRB number: 140315), the data from patients underwent PCNL at Changhua Christian Hospital were collected analyzed. Percutaneous nephrolithotomy was first introduced at Changhua Christian Hospital in 1987. Since 2009, the Urology department averaged around 150 PCNL per year has 8 board certified urologist and performed the procedure. From January 2010 to December 2014, a single urologist (MYY) performed one-stage fluoroscopic-guide percutaneous nephrolithotomy for complex renal and upper ureteral stone on 118 consecutive patients. We define complex renal stone as partial or complete staghorn stone, multiple renal stones in more than 2 calyxes, obstructive uretero-pelvic stone > 2 cm, and a renal stone in single functional kidney. Surgical indications were renal staghorn stones, large renal calculi (larger diameter > 2 cm), large upper ureteral stone (transverse diameter > 1.5 cm) or mixed. The decision on either supra-costal or sub-costal approach will be decided after intra-operative injection of contrast through retrograde ureter catheter. We usually choose puncture site that would result in maximum stone clearance and ease of double-J insertion in mind. If the desired entry point into the collecting system is feasible in subcostal, then subcostal approach is chosen and vice versa. All the patient received double-J ureteral stent. The decision to use nephrostomy was made at the end of the procedure. Exclusion criteria for tubeless procedure included: significant postoperative bleeding, significant perforation of the collecting system, much residue stone burden, multiple percutaneous tracts and obstructive renal anatomy. Patients were informed about the decision making prior to agreeing on undertaking the procedure. Of the 118 patients, eighty-six patients underwent tubeless percutaneous nephrolithotomy during the study period. Of the 86 tubeless cases, fifty-six patients underwent supra-costal approach and 30 patients underwent standard sub-costal approach. If the patients experience intrathoracic complication during the procedure, a pigtail drain would be inserted at the end of the procedure and would not defer from tubeless procedure. Fortunately, none of the patients experienced intrathoracic complications during the study period. Stone-free is defined as no visible stone at end of procedure taken with intraoperative fluoroscopy or stone ≤2 mm at follow-up KUB imaging. If large residual stone ≥20 mm which require staged operation, a nephrostomy tube would also be placed. If there is residual symptomatic (ie. hydronephrosis, renal colic pain, hematuria, etc) stone ≥5 mm, adjuvant treatment with ureteroscopic lithotripsy or extracorporeal shockwave were used.

### Pre-operative survey, operative method, and post-operative care

Preoperative evaluation of patients includes urine analysis, urine culture, serum creatinine, a kidneys ureter and bladder (KUB) X-ray, renal ultrasonography and intravenous pyelogram. Prophylactic antibiotics (1000 mg of cefazolin) was administered 30 min prior to the start of the operation, unless the patient is allergic to cephalosporine then alternative antibiotics would be used. All the procedure is performed under general anesthesia. With the patient in the lithotomy position, a 5.0 Fr. ureteral catheter was placed in the ipsilateral renal pelvis and secured on to the Foley’s catheter. The patient was then changed to the prone position, with all the pressure points protected with padding. Contrast medium was used to opacify the calyceal system via the ureteral catheter or a Chiba needle under ultrasonic guidance puncture. The “eye-of-the-needle” technique, as described by Dr. Arthur Smith, was used in establishing percutaneous access [17]. Most of the time, upper or middle post calyx is chosen for puncture under fluoroscopic guidance. After selecting the suitable calyx for puncture, a small 0.5 cm incision was made at the skin to help facilitate the insertion of the puncture needle. Once the puncture needle enters the collecting system and confirmed with fluoroscopy, a 0.038 in. guide wire is then passed into the collecting system and whenever possible into the renal pelvis or into the ureter. The skin incision is then extended to 1 cm and the nephrostomy tract is then dilated using Amplatz fascia dilator (Microvasive, Natick, MA, USA) until 26 Fr. diameter. A 26 Fr. access sheath is then placed in the collecting system and a 24 Fr. nephroscope (Richard Wolf GmbH, Knittlingen, Germany) coupled with ultrasonic lithotripter was used for stone fragmentation. The stone fragments were then removed using a 3-clawed forceps or suction. After all the visible stone is removed, a 6 Fr. double-J stent is placed in antegrade fashion for all the patients. In order to check for feasibility of tubeless cases, a guidewire in the collecting system then the access sheath is slowly removed while the nephroscope inspect the nephrostomy tract for any pulsating bleeders. After completely removing the access sheath, we further observe for pulsating or excessive bleeding from the nephrostomy tract. If there is pulsating or excessive bleeding, the access sheath is inserted into the collecting system with help of guidewire and a nephrostomy tube is inserted, otherwise the wound is closed with 2–0 nylon suture with a pressure dressing. Patients will start oral intake as soon as possible with the use of diclofenac 25 mg 3 times daily as oral analgesia if eGFR > 60. For patients with eGFR < 60, we will prescribe acetaminophen 500 mg 4 times daily. If the pain persists, intravenous pethidine 50 mg every 6 h pro re nata will be used for further pain control and the amount of intravenous analgesia would be recorded and analyzed. Cefazolin would be used up to 3 days as post-operative antibiotics. The Foley’s catheter is removed on post-operative day 1 and patients were on the average discharged on post-operative day 4 depending on their conditions. All patients were assessed with renal ultrasonography, KUB and CXR before discharge to confirm stone-free status and exclude the presence of urinoma or perirenal hematoma and hemothorax or pneumothorax before discharge. Double-J stents were removed 2 weeks after the operation. KUB and renal sonography will be arranged 1 month after the operation during clinic hours.

### Statistical analysis

Morbidity, operation time, analgesics requirement, length of hospital stay, stone- free rate, were analyzed. Statistical analysis was done using 1-way ANOVA, Pool t test and Chi-Square test, with *p* < 0.05 considered statistically significant. Calculations were performed using commercial software (JMP 6).

## Results

Thirty-two cases did not receive tubeless treatment and were excluded from our study. Eighteen cases had large residual stone burden, 10 had excessive nephrostomy tract bleeding, 1 underwent multiple percutaneous tracts, 2 underwent bilateral PCNL on the same day and 1 experienced pelvis perforation. A total of 86 tubeless cases (56 in supra-costal group and 30 in sub-costal group) were included in this study. The mean age, operation side, stone locations were similar in both groups. The male to female ratio is higher in supra-costal group (39/17) than in sub-costal group (13/17) (*p* = 0.0174). Large renal stones and staghorn stones occupied most of the stone cases (supra-costal group 75%, sub-costal group 80%) (Table 1). The mean operation time is 100 min in supra-costal group and 110 min in sub-costal group (Table 2). Stone location is related to the operative time with upper ureter stone being the shortest and staghorn stone being the longest (Table 3). Upper and middle calyx were the main entry sites in both groups. The initial stone-free rate is higher in the supra-costal group 59% (33/56) when compared to sub-costal group was 50% (15/30) (*p* = 0.4274) with the overall stone-free rate was 56%. All non-stone free patients will undertake post-operative ancillary procedures (extracorporeal shockwave lithotripsy or ureteroscopic lithotripsy) 3 months later, the total stone-free rate increased to 90% (Table 2). Upper ureteral stone group had the highest initial stone-free rate (10 out of 11 patients) and the staghorn stone group being the lowest (3 out of 25 patients, 1 in supra-costal and 2 in sub-costal) (*p* < 0.0001) (Table 4). Mean length of stay is similar in both group (4 days). There was no statistically significant difference in use of post-operative intravenous analgesia requirements (supra-costal 25.76 mg, sub-costal 33.92 mg) and hematocrit change (supra-costal 3.5%, sub-costal 3.3%) (Table 2). The overall complication rate is 6% including 1 patient supra-costal group (2%) and 4 patients in sub-costal group (13%) (*p* = 0.0292, Table 2). One patient in the supra-costal group was transferred to intensive care unit due to sepsis with respiratory failure. The complications in the sub-costal group includes three patients with acute pyelonephritis and one patient needing blood transfusion. All patients with complications recovered uneventfully (Table 5). Post-operative renal ultrasonography did not show evidence of perirenal hematoma or fluid accumulation.

**Table 1:** Patients Demographics profile

	Supra-costal(*n* = 56)	Subcostal(*n* = 30)	Total(*n* = 86)	
	No. (%)	No. (%)	No. (%)	*P* value
Sex				
F	17 (30)	17 (57)	34 (40)	0.0174
M	39 (70)	13 (43)	52 (60)	
Age(years)^a^	52.33 ± 11.75	55.43 ± 12.59		
Side				
Left	26 (46)	12 (40)	38 (44)	0.5672
Right	30 (54)	18 (60)	48 (56)	
Stone location				
Renal+upper ureter	8 (14)	1 (3)	9 (10)	0.4159
Renal	26 (46)	15 (50)	41 (48)	
Staghorn	16 (29)	9 (30)	25 (29)	
Upper ureter	6 (11)	5 (17)	11 (13)	
Stone Burden				
Length (mm)	42.59 ± 19.76	33.41 ± 18.00	38.73 ± 19.42	0.077
Width (mm)	27.03 ± 12.28	24.38 ± 13.98	25.92 ± 12.97	0.577
Dimension (L x W)	1346.51 ± 1111.07	999.46 ± 1166.55	1200.38 ± 1137.63	0.259

1. Chi-square test

2. ^a^Pool t-test

**Table 2:** Operation Outcomes

	Supra-costal(*n* = 56)	Subcostal(*n* = 30)	Total(*n* = 86)	
	No. (%)	No. (%)	No. (%)	*P* value
Puncture calyx				
Lower	1 (2)	3 (10)	4 (5)	0.2195
Middle	31 (55)	16 (53)	47 (55)	
Upper	24 (43)	11 (37)	35 (41)	
Op time(mins)^a^	100.71 ± 23.46	110 ± 27.38		
LOS (days)^a^	4.03 ± 2.33	4 ± 0.94		
Pethidine(mg) required^a^	25.76 ± 42.02	33.92 ± 56.60		
HCT change (%)^a^	3.53 ± 2.36	3.36 ± 2.40		
Stone-free (post op)				
No	23 (41)	15 (50)	38 (44)	0.4274
Yes	33 (59)	15 (50)	48 (56)	
Stone-free (3 months)				
No	6 (11)	3 (10)	9 (10)	0.9179
Yes	50 (89)	27 (90)	77 (90)	
Ancillary procedures	23 (41)	15 (50)		
Complications				
No	55 (98)	26 (87)	81 (94)	0.0292
Yes	1 (2)	4 (13)	5 (6)	

1. Chi-square test

2. ^a^Pool t-test

**Table 3:** Stone Location and Operation Time(minutes)

Level	Number	Mean	Std Dev	Std Err Mean	Lower 95%	Upper 95%
Renal+upper ureter	9	90	17.5	5.83	76.55	103.45
Renal	41	98.29	20.26	3.16	91.9	104.69
Stghorn	25	121.6	27.90	5.58	110.08	133.12
Upper ureter	11	96.36	21.57	6.50	81.87	110.86

1. Chi-square test, *p* < 0.0001

**Table 4:** Stone Location and Results Analysis

	Renal + upper ureter No. (%)	Renal No.(%)	Staghorn No. (%)	Upper ureter No. (%)	Total (*n* = 86) No. (%)	*P* value
Sex						
F	1 (11)	14 (34)	16 (64)	3 (27)	34 (40)	0.0148
M	8 (89)	27 (66)	9 (36)	8 (73)	52 (60)	
Side						
Left	4 (44)	18 (44)	12 (48)	4 (36)	38 (44)	0.9357
Right	5 (56)	23 (56)	13 (52)	7 (64)	48 (56)	
Puncture calyx						
Lower	0 (0)	2 (5)	2 (8)	0 (0)	4 (5)	0.3119
Middle	3 (33)	20 (49)	17 (68)	7 (64)	47 (55)	
Upper	6 (67)	19 (46)	6 (24)	4 (36)	35 (41)	
Operation-access						
Supra-costal	8 (89)	26 (63)	16 (64)	6 (55)	56 (65)	0.4159
Subcostal	1 (11)	15 (37)	9 (36)	5 (45)	30 (35)	
Stone-free						
No	1 (11)	14 (34)	22 (88)	1 (9)	38 (44)	< 0.0001
Yes	8 (89)	27 (66)	3 (12)	10 (91)	48 (56)	
Complication						
No	8 (89)	39 (95)	23 (92)	11 (100)	81 (94)	0.6999
Yes	1 (11)	2 (5)	2 (8)	0 (0)	5 (6)	

1. Chi-square test

**Table 5:** Comorbidities

Supra-costal (*n* = 1)		
Clavien-Dindo Classifications	number	
Grade 4	1	ICU admission due to sepsis with respiratory failure
Sub-costal (*n* = 4)		
Clavien-Dindo Classifications	number	
Grade 2	4	Pyelonephritis [3], blood transfusion [1]

## Discussion

Percutaneous renal surgery is a useful tool for urologists in treating conditions in the upper urinary tract. For complex renal stone, PCNL is as effective as open operation but with less post-operative discomfort and a shorter hospital stay. An optimal and atraumatic access to the desired calyx is the first step in a successful PCNL. In most cases, sub-costal puncture is preferred access; however, an upper pole access (via supra-costal area) is favored in cases of complex proximal. The advantage of upper pole over lower pole access is direct access to all calyces and the upper ureter but at a cost of increase risk of intrathoracic complications. In a study by Hopper & Yakes, percutaneous nephrostomy puncture in the intercostal space between the 11th–12th rib result in a lung injury in 14 to 29% of the patients while a 10th–11th rib intercostal space puncture result in lung injury in 86 to 93% of patients. After careful inspection of pleura anatomy, we noticed that the lowest point of the costo-diaphragmatic recess is at the medial half of the 12th rib, therefore an intercostal puncture on the lateral half 12th rib will less likely result in a punctured pleura. In cases of large complex renal stone, upper posterior calyx is the preferred access point to obtain maximum stone clearance [12, 18]. Due to the anatomical restrictions, supra-costal puncture is necessary for an adequate access into the upper posterior calyx. The stone–free rate in our series for supra-costal group was 59% (33/56) compared to 50% (15/30) in the sub-costal group (*p* = 0.4274). Since our series comprised of mostly large renal stone and staghorn stone, our overall stone-free rate of 56.9% is similar to CROES data for staghorn patients [19]. The reasons for initial low stone-free rate in our series compared to other tubeless studies include higher percentage of staghorn stone (77%), single nephrostomy tract, and use of rigid nephoscopy [10, 16, 19]. However, the use of post ancillary procedures improved the stone-free rate at 3 months to 90%. Sub-analysis showed that patients with proximal ureter stone is more prone to be stone-free due to smaller size. Length of stay and analgesic requirement did not have statistically significant difference between our supra-costal and sub-costal group, but the average amount of analgesia is less in supra-costal group (25 mg vs. 34 mg).

In the late 1980’s to early 2000, several studies report contradictory results about “tubeless” PCNL. Placement of nephrostomy tube at the end of PCNL procedure is routine for most urologist to assist renal healing, avoid urine extravasation, aid hemostasis and future access in staged procedures [20]. However, nephrostomy tube is associated post-operative pain and discomfort, analgesic use, and urine leak from nephrostomy tract [9, 21]. In 1997 Bellman et al. started using the term “tubeless” PCNL, the study included fifty patients underwent PCNL procedures with only internal double-J stent. In their series, tubeless PCNL resulted in lower length of stay (LOS) and less analgesic use with faster return to normal activity when compared to the standard PCNL [3]. In subsequent studies comparing to standard PCNL, the safety and efficacy of tubeless PCNL is confirmed with similar morbidities, while offering shorter LOS and less analgesic use [3–10, 15, 22, 23].

Fever and bleeding are the most common complications associated with percutaneous renal surgery. The overall complication rate in our study is 6% (5 of 86 patients), with infection being the most common (Table 5). The combination of pre-operative urine culture and prophylactic antibiotics helped manage post-operative infection without resulting in any mortality during our study period. Post-PCNL bleeding can arise from a variety of sources such as the collecting system, renal parenchyma, arteriovenous fistula, pseudo-aneurysm, or the intercostal or subcutaneous vessels. The most frequent source of bleeding after PCNL seems to be from the renal parenchyma-collecting system junction. In our opinion, a careful calyx selection and puncture angle is very essential in minimizing post-PCNL bleeding. Since most of our puncture site are either middle or upper calyx, supra-costal approach into the posterior calyx will ensure a more direct angle into the desired calyx through the avascular plane of Brodel that is parallel with the minor calyx. A punctured tract parallel with minor calyx will minimize injury to interlobar vessels. In contrast for sub-costal puncture to reach middle or upper calyx, the puncture angle would be wider and not as parallel as the supra-costal puncture which results in higher chance of injuring interlobular vessels. Only one patient (sub-costal puncture) in our current series experienced severe bleeding which required blood transfusion.

In order to minimize post-PCNL bleeding, several studies investigate different hemostatic methods and agents. Noller et al. reported the use of fibrin sealant in renal parenchyma defect which resulted in average 2% decrease of hematocrit and no patients needing blood transfusion [24]. Hemostatic agents such as gelatin, Surgicel (oxidized cellulose), and Tisseel sealants were also investigated and showed contradictory results [15, 22]. Jou et al. investigated the use of electrocauterization to stop bleeder in the PCNL tracts in 249 patients, which results in 84 patients (34%) not needing a nephrostomy tube [25]. In our series, no additional hemostatic agents or electrocauterization over nephrostomy tracts were used. Instead, we gauge the amount of bleeding in the nephrostomy tract while retracting the nephroscope to determine the necessity of nephrostomy tube. In our series, the average hematocrit decrease was 3.53% ± 2.36% (supra-costal) and 3.36% ± 2.40% (sub-costal). Our study shows the initial puncture selection is more crucial in developing post-PCNL bleeding than the use of other adjuvant hemostatic agents or electrocautery.

In Munver’s series of 300 percutaneous renal surgery, the overall complication rate was 8.3% (16.3% for supra-costal and 4.5% for sub-costal access) with supra-costal access having the most intrathoracic complications [13]. In our study, we did not experience any intrathoracic complications in the supra-costal (11th -12th intercostal) access group. Low incidence of intrathoracic injury can be attributed to careful review of anatomy and puncture selection. A careful anatomy puncture on the lateral half of 12th rib is a key in preventing intrathoracic injury. Limitations of our study include small study population and non-randomizing between the study groups.

## Conclusions

Traditionally, a supra-costal access is associated with significantly higher intrathoracic complication rates compared to sub-costal access in standard PCNL. From the low complication rate in our current study, tubeless PCNL is a safe and effective procedure in selected patients. With careful anatomical position and an experienced operator, tubeless PCNL with supra-costal puncture within the 11th and 12th intercostal space is not associated with increase intrathoracic complication or morbidity.

## Abbreviations

CROES: Clinical Research Office of the Endourological Society
DM: Diabetes mellitus
ESWL: Extracorporeal shockwave lithotripsy
IRB: Institutional review board
KUB: Kidney, Ureter, Bladder X-ray
PCNL: Percutaneous nephrolithotomy
WBC: White blood cells
